# Using conversation analysis to inform role play and simulated interaction in communications skills training for healthcare professionals: identifying avenues for further development through a scoping review

**DOI:** 10.1186/s12909-018-1381-1

**Published:** 2018-11-19

**Authors:** Alison Pilnick, Diane Trusson, Suzanne Beeke, Rebecca O’Brien, Sarah Goldberg, Rowan H. Harwood

**Affiliations:** 1 School of Sociology and Social Policy, University of Nottingham, Nottingham, NG7 2RD UK; 2 Institute for Mental Health, University of Nottingham, Nottingham, UK; 3 Language and Cognition Research Department, University College London, London, UK; 4 School of Health Sciences, University of Nottingham, Nottingham, UK; 5 Nottingham University Hospitals NHS Trust, Nottingham, UK

**Keywords:** Role play, Simulated patient, Simulated interaction, Communications skills training, Conversation analysis

## Abstract

**Background:**

This paper responds to previously published debate in this journal around the use of sociolinguistic methods in communication skills training (CST), which has raised the significant question of how far consultations with simulated patients reflect real clinical encounters. This debate concluded with a suggestion that sociolinguistic methods offer an alternative analytic lens for evaluating CST. We demonstrate here that the utility of sociolinguistic methods in CST is not limited to critique, but also presents an important tool for development and delivery.

**Methods:**

Following a scoping review of the use of role play and simulated interaction in CST for healthcare professionals, we consider the use of the specific sociolinguistic approach of conversation analysis (CA), which has been applied to the study of health communication in a wide range of settings, as well as to the development of training.

**Discussion:**

Role play and simulated interaction have been criticised by both clinicians and sociolinguists for a lack of authenticity as compared to real life interactions. However they contain a number of aspects which healthcare professionals report finding particularly useful: the need to think on one’s feet in real time, as in actual interaction with patients; the ability to receive feedback on the simulation; and the ability to watch and reflect on how others approach the same simulation task in real time. Since sociolinguistic approaches can help to identify inauthenticity in role play and simulation, they can also be used to improve authenticity. Analysis of real-life interactions using sociolinguistic methods, and CA in particular, can identify actual interactional practices that are used by particular patient groups. These practices can then be used to inform the training of actors simulating patients. In addition, the emphasis of CA on talk as joint activity means that proper account can be taken of the way in which simulated interaction is co-constructed between simulator and trainee.

**Summary:**

We suggest that as well as identifying potential weaknesses in current role play and simulation practice, conversation analysis offers the potential to enhance and develop the authenticity of these training methods.

## Background

This paper responds to previous debate published in this journal around the use of sociolinguistic methods in communication skills training (CST) [1], which has raised the significant question of how far consultations with simulated patients reflect real clinical encounters. The previous debate concluded with a suggestion that sociolinguistic methods offer an alternative analytic lens for *evaluating* CST. We extend this argument by suggesting that a specific sociolinguistic approach, conversation analysis (CA), also presents an important means of *developing and delivering* CST. Following a scoping review of the use of role play and simulated interaction, we focus on CA, which has a growing evidence base from its application to the study of health communication in a wide range of settings [2, 3] as well as to the development of training [4]. CA is used to study the structure and organisation of talk, and to identify patterns in real-life communication encounters. It analyses what communication partners actually do, rather then what they think or say they do. The underpinning principle is that interaction is a joint activity that is co-constructed by all participants [5]. We suggest that as well as identifying potential weaknesses in current role play and simulation practice, CA offers the potential to enhance and develop the authenticity of these training methods.

Role play and simulation are both experiential learning methods which incorporate the creation of a scenario in which skills can be rehearsed. Whilst often used interchangeably, there is an important distinction. In this paper ‘role play’ will be used to refer to training in which the learner themself is required to take on a different character (for example a patient). Simulation will be used to describe training where external actors, who have been trained in simulation techniques, take on the patient role.

## Communication skills for healthcare professionals

The importance of effective communication skills in healthcare is well-documented. Healthcare professionals (HCPs) need skills to be able to gather personal information, often from an anxious patient. They need to impart information sensitively [6], including breaking bad news [7], and they may need to gain informed consent and co-operation for medical procedures [8], or motivate healthy behaviour [9]. Failure to communicate effectively can lead to breakdown in the therapeutic relationship, patient dissatisfaction and can affect patient outcomes [10]. Communication skills are part of the General Medical Council’s requirements for all medical school curricula [11], and also a key part of pre-registration nurse education [12]. However, communication between HCPs and patients remains challenging. For example, medical jargon familiar to HCPs may be a barrier to patient understanding, or the distress of receiving an unwelcome diagnosis or bad news may inhibit patient understanding or retention of the information communicated. In addition, lack of effective communication skills can lead to physicians’ avoidance of discussing difficult issues [10]. Communication is further complicated with the significant number of patients who have communication impairments such as those who are living with dementia, aphasia associated with a stroke, learning disabilities or hearing loss. HCPs may have little knowledge of the communication abilities of the patient before they start the conversation.

It follows from the above that all HCPs need training in how to inform patients about their disease/condition and treatments, and how to create a therapeutic relationship through empathy, concern and providing comfort and support [13]. Improving HCP communication is an NHS priority. The role of communication in reducing errors and improving patient care has been underlined by the Bristol Royal Infirmary Inquiry [14], and the report ‘Building a Safer NHS for Patients’ [15], both of which recommend additional communication skills training for HCPs. The national core standards for healthcare professionals in England specifically reference communication and interaction skills [16]. However, healthcare professionals report they lack confidence and need more communication skills training, particularly with patients they perceive as challenging [17]. Evidence suggests this training is necessary since communication skills cannot be improved through clinical experience alone [18]. Additionally, since communication cannot be entirely predictable in practice, and requires decisions and responses in real time, simulation and role play are common techniques which are incorporated into this training.

## Literature review: Search strategy

Scoping reviews are a technique used to map relevant literature in a field; they do not generally seek to address very specific research questions or assess the quality of included studies [19]. Our purpose here was in line with one of the four common purposes of a scoping review: to identify research gaps in the existing literature on the incorporation of role play and simulated patients in training healthcare professionals, and to build on this by drawing conclusions about future directions [19]. The search strategy involved the following databases: PubMed, Scopus, and Web of Science. We used the search terms ‘simulated interaction’, ‘simulated patient’, ‘role play’, and ‘conversation analysis’. We used the term simulated patient rather than ‘standardized patient’ to reflect the fact that the latter term is less commonly used outside North America, and that the 2017 Association of Standard Patient Educators’ Standards of Best Practice update states that ‘simulated patient’ is the more inclusive term to refer to all human role players in any simulation context (https://www.aspeducators.org/about-aspe). These search terms were used in combination with the terms ‘communication skills’, ‘training’ and ‘healthcare professional’. Articles from 2011 onward were searched; this date was chosen because it represents a landmark in the formal documentation of the emerging use of CA in the development of training interventions [20]. Abstracts and titles were screened for relevance. Articles were included if they described empirical studies of communication skills training for healthcare professionals involving simulated interaction or role play, including qualitative, quantitative, and mixed methods. In total 925 studies were identified from the search, and 897 were excluded, leaving 36. Exclusions commonly described the use of mannequins or simulation in clinical procedures (e.g. simulated surgery) as opposed to interactions with simulated patients, or were duplicates.

Relevant articles were reviewed in full, with a focus on the methodology used and evaluation of the interventions, using a template to summarise the main points. As per scoping review guidelines, reference lists of the included publications were also scrutinised, and our existing networks were consulted, leading to the inclusion of 12 additional studies published prior to 2011. The review was conducted iteratively, first examining those studies reporting working with role play or simulated patients, and then focusing specifically on the use of conversation analysis to inform CST in healthcare settings.

## Role play

Proponents of role play argue that it promotes active learning, enabling participants to have concrete experiences and reflect on those experiences from their own and other people’s perspectives [21]. Role play when used as an assessment tool has been posited as a way of achieving a more natural conversation or realistic interaction, because it disrupts the usual power imbalances between assessed and assessor [22], though this will be context dependent.

Role play has been assessed in the training of medical [23–26] and nursing students [27]. These studies report successes: an increase in empathy [23, 24]; an opportunity to practice communication skills in a safe and supportive environment [23]; an opportunity to experience more unusual clinical scenarios [24]; the value of peer feedback [26, 27]; and (in cases where the role play was video recorded) the opportunity to observe one’s own body language and interaction [27]. However, students also have reservations about the process, noting the artificiality of the scenario [13, 26] and the potential lack of authenticity due to poor acting skills on the part of the role players [26].

The choice of role play with student groups is likely to reflect limited resources in these settings. However, it raises particular questions over authenticity, to be considered further below. It also does not shed any light on the acceptability or success of this approach with non-student populations (i.e. practising doctors or nurses), or other groups of healthcare professionals.

## Interactions using simulated patients

Although role play with peers can be an easily arranged and cheap way of teaching communication skills, actors who have undergone specialised training are increasingly worked with to portray patients with various health conditions in training and assessment. It has been argued that these standardised or simulated patients (SPs) ‘can provide realistic presentations of patients with specified conditions to allow students to experience the real sense of treating a patient’ ([28] p.513), which encourages students to seek their own solutions rather than passively taking in information. The aim is that a sense of realism is brought to clinical learning, compared to hypothetical, paper-and-pencil approaches to problem-based learning.

Two reviews of studies where learners engaged with SPs [29, 30] suggests a number of advantages for training and assessment compared to trainees’ role- playing. For instance, they are able to come out of their role to provide feedback about trainees’ strengths and weaknesses, from the ‘patient’s’ perspective; they are also able to do so with professional competence and consistency [31, 32]. It is also claimed that using SPs in training can be more effective than peer role-play, because the SP is unknown to the trainees which adds authenticity to the encounter.

Empirical studies have highlighted the benefits of using SPs when assessing communication skills. Studies have been conducted in a range of settings, with student trainees [10, 33–36] and with practicing healthcare professionals including doctors [7, 10] and wider healthcare teams [37]. Clinical specialisms have included anaesthesia [8], obstetrics [37], emergency medicine [10], speech and language difficulties [33] and psychiatry [34, 35]. A broad range of communication skills have been assessed through these studies including the taking of consent [8], breaking bad news [7] and conducting cognitive language assessments [33].

Taken as a group, all these studies report positive effects. These include: increased confidence of the students or HCPs [34, 36]; improved communication performance as judged by the simulated patients [33, 37]; and improved performance in standardised assessments [7]. Findings suggest that CST training using simulated patients was appreciated and valued, particularly for the ‘safe’ environment it provided in which trainees could practice skills. Only one study reports any negative effects, highlighting two students from a first-year medical student cohort who reported decreased confidence following their training, with a realisation of how much they still had to learn [36]. However, this is likely to be a short-term negative consequence which can be turned into a longer term positive.

In addition to the individual studies described above, a recent review of studies using SPs in CST for nurses reported improvements in nursing students’ confidence and communications skills in interventions where they engaged with SPs [38]. However, the authors noted that SPs are typically worked with in scenarios where communication is particularly challenging (for example breaking bad news). They concluded by arguing for expanding the range of utilisation, but also for an increased methodological rigour in using, and studying the use of, SPs.

## Strengths and limitations of using simulated patients

Simulated patients were worked with in five of the 14 studies contained in a review of nurses’ communication training [6]. The authors of the review argue that using simulated patients is advantageous because they can play a role consistently with every trainee. However, they acknowledge that simulations can be perceived as an artificial encounter and ‘real’ patient outcomes cannot be measured when using SPs. Using real patients is more authentic but individual differences make outcomes difficult to measure (for research, selection or competency assessment), and is not always appropriate or practical. The authors therefore propose using a combination of real and simulated patients in training, although this approach was not found in any of the studies that they reviewed. However, our review found two studies where the approaches were directly compared. The first compared the application of a generic communication skills training programme with standardised vs real patients [39]. The trainees were a mixed group of HCPs. Improvements in communication were observed in all but one training group, and differences between the observed skills used across standardised and real samples were non-significant, suggesting skills transfer from simulation to actual practice. The second study compared student-reported communications skills, knowledge and confidence across standardised patient, virtual and traditional learning environments [40]. Their trainees were UG students, who experienced either an actor playing a patient, a virtual patient (computer simulation), or a real patient in a nursing home. Higher communications skills, knowledge and confidence were reported across all three settings, with no significant differences. The authors use the findings to argue for a greater role in CST for virtual patients, but the results also show that engagement with SPs was as effective as ‘real’ patients in this context.

Work has also been done to directly compare the effects of using peer role play vs using simulated patients in the same interventional context, in a study of experienced medical practitioners who were being trained in behaviour change counselling [9]. Trainees were randomly assigned to either a group using a SP, or a group doing role play with each other. All 70 participants had standardised consultations with a SP before and after training which were assessed by observers using a Behaviour Change Counselling Index rating scale. Results revealed that trainees reached the same level of competence in both the experimental and control groups, with the authors concluding that experiential learning is beneficial, whether this is with SPs or peers. One interpretation of these findings is that role play between experienced practitioners can be a more cost-effective way of CST. However, as the authors noted, all the trainees on the course were self-selecting; they were also experienced health practitioners and this is likely to have had an impact on the quality of the role play which may not be replicated with student or other less experienced populations. Other studies comparing SPs with peer role play have found higher levels of practical clinical skills in the SP group compared with the role play group [41], alongside higher levels of satisfaction from trainees [42].

However, while there are reported advantages of working with SPs over role play, there are still reported disadvantages as compared to real patients. In one study, medical students reported that SPs provided more detailed feedback on their communication skills than real patients, which improved the students’ confidence [43]. However, they admitted to doing less preparation for interactions with SPs and reported that they found real patients to be more willing to give information without constant prompting. After dealing with real patients, some students became conscious of the ‘performance’ aspects of SP interactions. Nevertheless, they valued the safe environment of these encounters in preparation for clinical practice. The authors conclude that SPs are valuable in teaching medical students and their benefits should be maximised, but that there is scope for improvement in the authenticity of the training provision.

## Increasing authenticity in simulation

Whilst most evidence suggests that working with SPs is advantageous in CST for healthcare professionals, who plays the role of SPs and how they are trained can impact on the perceived authenticity of the simulation.

Some authors have suggested that using fellow professionals addresses issues around authenticity, as in a study where GP trainees were used as simulated patients instead of actors [44]. They argue that the GP trainees were able to bring their authentic experiences to the role, which would not be possible for non-medical actors, who could not be aware of the possible ideas, models and acronyms that might be used within a role play. However, some trainees felt uncomfortable with the process because they did not want to be confused with the role that they were enacting for the purposes of the simulation; for example a ‘difficult’ patient. Using HCPs in this way may therefore have an ongoing impact on staff relationships within a team or unit. It may also mean that issues such as the use of medical jargon or acronyms might not be problematised within an interaction.

Other authors have attempted to address this issue by ensuring that their SPs have significant training input from clinicians with expertise in the field. One study utilised a group of drama students, who received training from existing role players from the learning disabilities field of nursing [45]. Drama students being taught to act as SPs were taught skills based on Nursing and Midwifery Standards including building therapeutic relationships with patients with different capabilities and needs. Communication skills training (CST) began by outlining ground rules including the need for non-judgmental participation and constructive, positive feedback. This training was designed to support the drama students in relating to the situations they were being asked to portray and to be better able to give a layperson’s view of the medical interaction. For example they identified the use of jargon in the responses of nursing students and gave feedback on their emotional responses.

Whilst the above example relied on the expertise of practitioners to train simulated patients, other studies have drawn on patient experience to enhance authenticity. In some studies, real patients have worked with SPs to share their medical history to better reflect the ways that patients present in a genuine consultation [13, 46]. However, critics of existing approaches to simulation have argued that using real patients’ accounts may not in itself be enough to ensure authenticity because people tend to embellish their accounts of medical encounters [47]. The counter argument to this is that simulated patient methodology promotes the design of patient narratives around specific learning objectives, so that ‘real’ patient stories are always educationally translated for use in teaching [48]. However, the issue of interactional authenticity, in the sense that whilst interactions may cover the same clinical ground they may not cover the same interactional ground or incorporate the same interactional challenges, remains.

Some studies using actors have attempted to select actors with particular relevance to or experience of a specific setting, such as using actors with intellectual disabilities in simulated healthcare scenarios that might present in primary care or emergency services [49]. However, the authors point out that involving actors with intellectual disabilities may have drawbacks such as causing stress or anxiety for the actors, who may find it difficult to sustain the role of a simulated patient. This drawback may also be apparent in other patient groups. This tension, between the desire to reconcile the difficulties of a moral commitment to avoid suffering (hence reducing realism) with an aesthetic commitment to realistic portrayal, has been the subject of significant discussion in the literature on simulated patients [50].

## Critiques of role play and simulations in communication skills training

The general use of role play in communication skills training (CST) has been criticised by prominent sociolinguists [51]. This critique is based on work which uses conversation analysis (CA) to compare audio recordings of police interviews which used role play for assessment purposes with recordings of real police interviews, neither of which had originally been recorded for research purposes. Using CA to examine the interactions in both cases demonstrated substantial differences between real life interviews and those using role play [51], casting doubt on the assumptions that role play is an accurate representation of what happens in real life situations. For example, in role-played encounters the participants would interact before the ‘proper’ interview started and they used ‘in’ jokes. Also, role-played interactions were found to be exaggerated, suggesting that they were aimed at fulfilling the assessment criteria rather than being actions that would take place in actual interviews. For instance, in the role-played interview the suspect was invited to call the police officers by their first names, meeting the recommendation for ‘establishing rapport’, but this did not happen in the real interviews. These findings are used to raise concerns that role play is accepted unquestioningly for training and assessment purposes in a range of occupational settings, and the author calls for greater authenticity, particularly for training healthcare professionals [51].

Similar concerns were expressed in a study using conversation analysis to compare the transcripts of real and simulated surgical consultations [52]. Although similar elements were found in both encounters (history taking, diagnosis, treatment options), marked differences were found in the way that real patients and SPs presented their problems. In particular, real patients described their symptoms in the context of their previous illness experiences and their journey to the referral. In contrast, SPs presented their symptoms as ‘strange and unrecognizable’ with no references to the referral process to justify their visit to the consultant, as if they were primary care patients rather than patients who had been referred to a specialist. The authors argue that this may have implications for authenticity in simulated interactions aimed at improving communications between HCPs and patients in their care. In identifying these implications, the authors are not critical of individual SP practice, and it might be argued that the ‘inauthenticities’ identified here could be resolved through the implementation of better educational practices in simulation. However, these findings point once again to the difficulty of achieving interactional authenticity alongside clinical authenticity.

Differences were also found when conversations between student HCPs and SPs were analysed using discourse analysis [53]. SPs were identified as generally more dominant in the conversation than real patients would be, and also closings were initiated by the SP rather than the HCP. Consequently, the authors suggest that it is not possible for simulated interactions to be completely authentic because they are removed from normal contexts. The complexity of playing multiple roles within the interaction was also highlighted as possibly limiting authenticity. For example, the student could be seen to be playing both trainee and HCP roles; the SP was creating opportunities for trainees to show their communication skills whilst at the same time portraying a realistic patient. However, they conclude that this apparent lack of authenticity should not be considered problematic. They argue that in a CST setting, the educational benefits should take precedence over aiming for authenticity.

## Alternatives to role play and simulated interaction: The use of conversation analysis

In response to concerns regarding the use of role play in CST, a novel approach has been developed using conversation analytic evidence: the ‘Conversation Analytical Role-play Method’, (CARM) [4, 54]. It is argued that this method, which uses videos of real-life (anonymised) examples of interactions, can benefit training in a range of situations; it has been used extensively in the field of mediation but the author argues that the principles are likely to be applicable in other areas of institutional interaction where role play and simulation are widely used pervasively.

The CARM method presents both transcribed and recorded conversations in real time. The basis of the method is to stop the recording of the conversation at certain points to allow trainees to discuss the ways that the conversation is either helping or impeding the preferred outcomes [54], and it is argued that despite having no prior knowledge of conversation analysis, workshop participants soon become oriented to the fine details of the interaction. Trainees are able to discern how the choice of words, pauses, silences, etc. could impact on the interaction between mediators and clients during telephone conversations. Consequently, they are able to learn ways to improve their communication skills in order to achieve desired outcomes. However, whilst the CARM method overcomes some of the inauthenticity of role play, it does remove some of the principles which HCPs report finding particularly useful: the need to think on one’s feet in real time, as in actual interaction with patients; the ability to receive feedback on the simulation; and the ability to watch and reflect on how others approach the same simulation task in real time. It is also possible that there will be differences in how much individuals in the groups contribute, which is not the case when doing a simulated exercise.

## Using CA to design role play/simulated patient interventions

The development of the CARM method links to a broader discussion over the role CA can play in the training of healthcare professionals. For example, CA has shown how the ways in which medical questions are posed can have different consequences for patients’ responses [55], and it has therefore been suggested that CA researchers can work with physicians to implement specific interventions in the ways that they interact with patients by changing their communication behaviour. Rather than use CA to develop a critique of simulation, or an alternative approach to training, we suggest that an alternative way forward is that CA could be used to *underpin* role play and simulation training.

Recognising the benefits of the CARM method has led some commentators to suggest that it could be developed by using transcriptions of medical encounters to identify a repertoire of conversational regularities which could be used to create authentic role play scenarios [47]. These regularities might include the ways that ‘patients ask questions in a consultation, how they place and time those questions in relation to the doctor’s actual communication behaviour, how patients respond to information delivery, how patients voice their information needs and participate in shared decision-making and generally how patients structure their involvement in the consultation process’ ([47]: p51). Such an approach is in keeping with findings from an international comparative case study of engagement with SPs [56]. The authors of this study refrain from making generic recommendations about best practice because they note the importance of contextualisation, e.g. to cover professional or disciplinary requirements. Grounding engagement with SPs in analysis of real interaction from the setting in question, alongside the incorporation of good simulation design principles, is arguably the most authentic way to achieve this contextualisation.

There are already the beginnings of such work in the literature which demonstrate the utility of CST interventions underpinned by CA. One such intervention, described as ‘inspired by’ the CARM method, aimed to help neurologists to distinguish between epileptic and nonepileptic seizures (NES) by identifying linguistic features in seizure patients’ talk [57]. Noting the importance of soliciting unconstrained narratives in the opening sections of the consultation, a one-day training program was developed to teach senior neurology trainees to design questions appropriately. By being encouraged to ask a neutral opening enquiry (‘so how can I help you today?’), and tolerate silences, there were marked improvements in eliciting narratives from patients that revealed diagnostically useful interactional and linguistic features when measured in video footage of real clinical encounters before and after training. The authors describe the effects of the one-day CA based intervention as having ‘exciting implications’ for facilitating diagnostic accuracy and possibly improving patient satisfaction.

CARM [4, 54] formalises the use of recorded naturally-occurring interactions as part of a training intervention. However, there has been a longstanding tradition of CA researchers working in healthcare making use of recorded materials as a training resource. One study used CA to assess the extent to which therapeutic relationships were enhanced by a training intervention for psychiatrists treating patients with psychosis [58]. The training intervention involved watching videos of interactions and suggesting alternative courses of action, which might better allow patients to describe the voices and hallucinations that they were experiencing. The aim was to acknowledge and achieve a shared understanding of these experiences, rather than avoid discussing them. The training also involved role play with each other and with SPs portraying patients with psychosis, in scenarios based on real interactions. In addition, psychiatrists had a ‘hearing voices’ exercise where they performed cognitive tasks whilst hearing voices through a personal audio (MP3) player. This was described as ‘very powerful’ in helping them to understand why patients wanted to talk about their voices.

Researchers analysed videos of psychiatrist/patient encounters before and after their intervention, identifying the number of self-repairs that occurred in the interaction. Self-repairs are described as conversational devices made by speakers to resolve problems in understanding and re-establish the flow in conversation [59]. The authors of the psychosis treatment intervention describe how self-repairs are a useful measure of how hard people are working to make themselves understood [58]. After the training, CA revealed more instances of self-repairs in the transcripts of psychiatrist/patient encounters, which the authors argued is indicative of the efforts that psychiatrists were making to achieve shared understanding of the patient’s symptoms. However, the psychiatrists were not trained specifically to use self-repair because of a desire to avoid introducing artificiality; rather, self-repairs happened naturally as a consequence of enhanced communication between psychiatrists and their patients. The authors claim this was the first intervention in mental healthcare to show improvements in communications skills in the treatment of psychosis. It also showed improvements in both the psychiatrists’ and their patients’ views of the therapeutic relationships. However, given the combination of methods that were used as part of the intervention, it is difficult to establish which elements were most effective, or indeed whether the result was achieved through the use of methods in combination.

CA has also played a role in training speech and language therapists (SLTs) to maximise the delivery of communication training for people with brain injury and their family members, with a particular focus on the language disorder aphasia, commonly caused by stroke. After early conversation analytic investigation of barriers and facilitators to interaction between people with aphasia (PWA), their peers and SLTs, CA has provided a tool both for guiding SLTs in delivering interventions, and for training PWA and their regular conversation partners to achieve mutual understanding in the face of conversation breakdown [60]. The authors highlight how encouraging patients and family members to watch videos of their own interactions and suggest alternative courses of action can be a powerful tool for behaviour change despite the presence of a language disorder. Subsequent studies evaluating the outcomes of a standard intervention package underpinned by CA, Better Conversations with Aphasia [61] have shown significant positive change in interaction for both PWA and their conversation partners [62, 63]. The BCA resource also includes online self-study for SLTs delivering the intervention. Using videos of the BCA intervention, activities encourage SLTs to reflect on the facilitative nature of neutral questions such as ‘what is happening here?’ to open video-feedback discussions with patients and family members around communication facilitators and barriers. This method appears to enhance patient and communication partner insights into the impact of certain interactional behaviours [64], and to encourage successful problem-solving around strategy use to enhance mutual understanding.

## Conclusion: Developing the use of CA to inform simulation

Participating in interactions with a SP displaying observed characteristics and responses of a particular patient group offers HCPs an opportunity to develop confidence and expertise in a safe and supportive environment, with no repercussions for actual patients [65]. This approach to simulated interaction also has the advantage of requiring the HCP to deal with situations in real time, as opposed to the considered reflection possible during CARM training, but not available to clinicians in ‘real life’. In addition, valuable learning can be gained from both being an observer and receiving feedback from observers. The opportunity to stop, start, rewind and replay the encounter is probably unique to simulated encounters; it would place too great a burden on real patients and would require acting skills beyond most HCPs in role play. Consequently, we argue that the use of CA to enhance real-time simulated interaction, rather than provide a substitute for it, presents a useful addition to the CST field. Previous research has used sociolinguistics to identify evidence for a gap between real interactions and assessed role plays [1], but we suggest here there is an important role for development as well as critique. The emphasis of CA on talk as joint activity means that proper account can be taken of the way in which simulated interaction is co-constructed between simulator and trainee.

There are potential disadvantages of using CA to develop simulated healthcare encounters in CST interventions in that it is labour intensive, requires at least one team member with expertise in CA and can be relatively costly in comparison to some other CST interventions [47]. However, the value of role play and simulated interaction are well established across a wide range of healthcare settings, and this approach has the advantage of offering a means to improve an already tried and tested approach.

## Abbreviations

BCA: Better Conversations with Aphasia
CA: Conversation Analysis
CARM: Conversation Analytic Role Play Method
CST: Communication Skills Training
PWA: People with Aphasia
SP: Simulated Patient
